# *NFKB2* mutation in common variable immunodeficiency and isolated adrenocorticotropic hormone deficiency: A case report and review of literature: Erratum

**DOI:** 10.1097/MD.0000000000008572

**Published:** 2017-11-03

In the article, “*NFKB2* mutation in common variable immunodeficiency and isolated adrenocorticotropic hormone deficiency: A case report and review of literature”^[1]^, which appeared in Volume 95, Issue 40 of *Medicine*, one of the references in Table 2 appeared incorrectly. The second Liu et al^[6]^ should have appeared as Lindsley et al^[3]^.

**Table 2:**
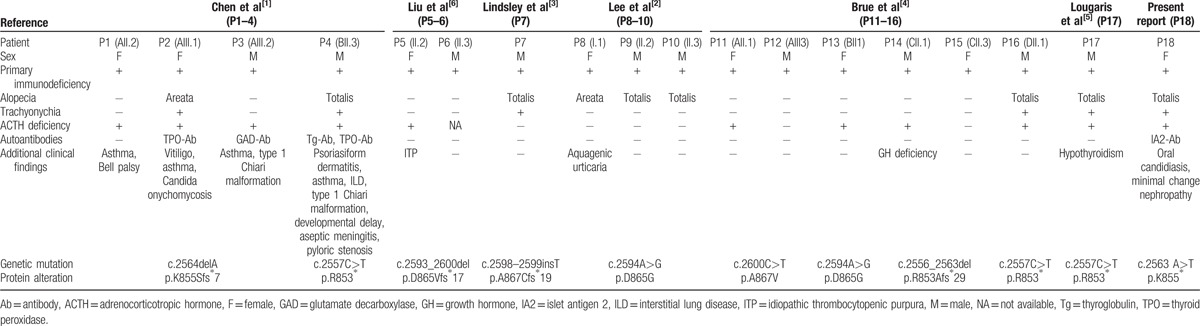
Reported cases with *NFKB2* mutation.

## References

[1] Shi C Wang F Tong A *NFKB2* mutation in common variable immunodeficiency and isolated adrenocorticotropic hormone deficiency: A case report and review of literature. *Medicine*. 2016 95:e5081. 27749582 10.1097/MD.0000000000005081 PMC5059085

